# Extracellular vesiculo-tubular structures associated with suberin deposition in plant cell walls

**DOI:** 10.1038/s41467-022-29110-0

**Published:** 2022-03-18

**Authors:** Damien De Bellis, Lothar Kalmbach, Peter Marhavy, Jean Daraspe, Niko Geldner, Marie Barberon

**Affiliations:** 1 Department of Plant Molecular Biology, DBMV, UNIL-Sorge, University of Lausanne, 1015 Lausanne, Switzerland; 2 Electron Microscopy Facility, University of Lausanne, 1015 Lausanne, Switzerland; 3 Department of Botany and Plant Biology, University of Geneva, 1211 Geneva, Switzerland; 4 Present Address: Sainsbury Laboratory University Cambridge, CB2 1LR Cambridge, United Kingdom; 5 Present Address: Department of Forest Genetics and Plant Physiology, 90736 Umeå, Sweden

**Keywords:** Plant cell biology, Plant development

## Abstract

Suberin is a fundamental plant biopolymer, found in protective tissues, such as seed coats, exodermis and endodermis of roots. Suberin is deposited in most suberizing cells in the form of lamellae just outside of the plasma membrane, below the primary cell wall. How monomeric suberin precursors, thought to be synthesized at the endoplasmic reticulum, are transported outside of the cell, for polymerization into suberin lamellae has remained obscure. Using electron-microscopy, we observed large numbers of extracellular vesiculo-tubular structures (EVs) to accumulate specifically in suberizing cells, in both chemically and cryo-fixed samples. EV presence correlates perfectly with root suberization and we could block suberin deposition and vesicle accumulation by affecting early, as well as late steps in the secretory pathway. Whereas many previous reports have described EVs in the context of biotic interactions, our results suggest a developmental role for extracellular vesicles in the formation of a major cell wall polymer.

## Abstract (web summary)

Suberizing plant cells export suberin monomers outside of the cell to form a hydrophobic barrier. Here the authors propose a role for extracellular vesiculo-tubular structures in the deposition of suberin monomers.

## Introduction

Extracellular vesicles or tubules (EVs) are nanosized membrane-encapsulated structures involved in the secretion of various molecular cargos including proteins, nucleic acids, metabolites and lipids^1^. A range of vesicles of different size and cellular origin including microvesicles (50-1000 nm) budding from the plasma membrane (PM) and exosomes (50-150 nm) derived from multivesicular body (MVB)-PM fusion are subsumed by the term EV. In animals, EVs release biomolecules into the extracellular space for targeted intercellular communication. In plants, EVs have been reported early on under various designations (paramural bodies, plasmalemmasomes or boundary structures) and speculated to be associated with cell wall synthesis^2–4^. In the last decades, EVs have been reported to contain RNAs, defense compounds and signaling lipids and are considered to play a central role in inter-organism communications during defense and symbiosis^5–11^. More recent data implicating EVs in cell wall formation and modification were mostly reported in the context of induced defense responses^11–14^. It is evident that any type of plant cell wall formation relies on a multitude of secreted molecular building blocks and enzymes for its construction^15^, yet little has been reported concerning the role for EVs in general cell wall formation during development. EV containing bodies (referred to as “paramural bodies”) were reported to be increased in vesicle trafficking mutants^16,17^, but it remains largely unknown whether EVs are involved in the regular deposition of cell wall polymers during plant growth and development.

Suberin is a major secondary cell wall formation in plants. In young, Arabidopsis primary roots, it occurs exclusively in the endodermis^18^. By using various genetic, as well as hormonal perturbations of suberin deposition, we demonstrate a strict association of bodies containing extracellular vesicular-tubular structures (EVBs) with suberin deposition in the cell wall. We were able to visualize EVBs not only upon chemical fixation, but also after high-pressure freezing, freeze substitution, excluding that they represent chemically induced fixation artifacts. Moreover, we demonstrate that inhibition of the secretory pathway at early and late stages interferes with both EVB formation and suberin accumulation, suggesting that EVBs are required for the transport of suberin precursors or biosynthetic enzymes to the apoplast and for the formation of this major secondary cell wall in plants.

## Results and discussion

### Extracellular vesiculo-tubular structures accumulate in endodermal barriers mutants

**Fig. 1:**
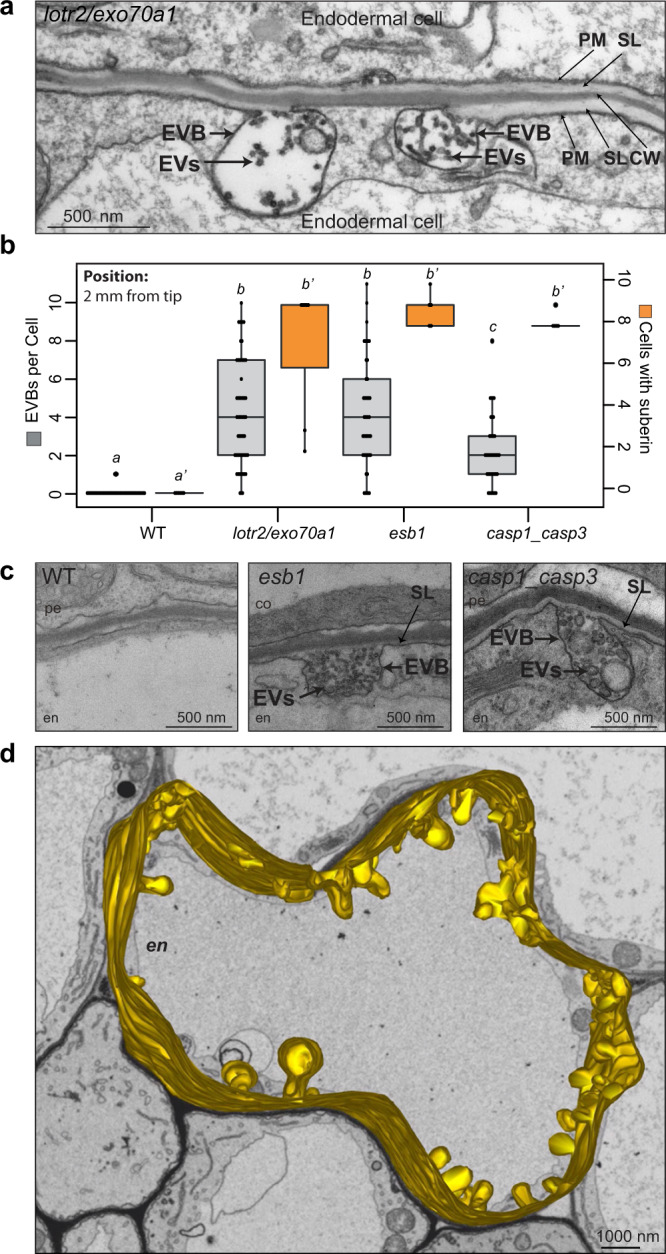
Bodies with extracellular, vesiculo-tubular membranes accumulate in endodermal barriers mutants. **a**, **c**, **d** TEM sections showing cell wall (**CW**), suberin lamellae (**SL**), plasma membrane (**PM**), bodies (**EVBs**) containing extracellular, vesiculo-tubular membranes (**EVs**) in endodermal (**en**) cells. **pe**, pericycle; **co**, cortex. **a** Endodermal section in *lotr2*/*exo70a1* mutant at 2 mm from root tip. Representative picture in a portion of 1 endodermal cell out of 7 individual roots from 3 independent experiments. **b** Number of EVBs (in grey, left axis) and number of suberized cells (in orange, right axis) in endodermal layers in TEM cross-sections of complete roots at 2 mm from root tip in WT, *lotr2*/*exo70a1*, *esb1* and *casp1_casp3* mutants (data for WT, *esb1*, *casp1_casp3* are also shown in Supplementary Fig. 3b). Data are presented as dot plots with box plots overlaid (*n* = 49, 61, 44, 41 endodermal cells for EVB number and *n* = 6, 7, 5, 5 root sections for number of suberized endodermal cells in WT, *lotr2*/*exo70a1*, *esb1* and *casp1_casp3* respectively). For boxplots, box shows the quartiles, whiskers indicate the minimum and maximum non-outlier values, dots located outside the whiskers of the box plots indicate outliers falling outside 1.5 times the interquartile range above the upper quartile and bellow the lower quartile, and center line corresponds to the median. Different letters indicate significant differences between genotypes or growth conditions (*P* < 0.05). **c** Endodermal sections in WT, *esb1,* and *casp1_casp3* mutants. Representative pictures in a portion of 1 endodermal cell out of 6, 8, 5 individual roots from 2, 4, 3 independent experiments for WT, *esb1* and *casp1_casp3* respectively (see also Supplementary Fig. 1a with more pictures for *esb1* and *casp1_casp3*). **d** 3D model of the PM and its EVBs (highlighted in yellow) in *lotr2*/*exo70a1* mutant. The model was done on a Z portion of 10 μm starting at 2 mm from the root tip (250 sections, 40 nm thick from a FIB-SEM stack in 1 representative endodermal cell from 1 root section out of 7 individual roots from 3 independent experiments analyzed (see also Supplementary Fig. 1b, c and Supplementary Movie 1).

In our efforts to understand endodermal differentiation, we performed several genetic screens for endodermal barrier mutants^19,20^. One screen identified the *lord of the rings 2* mutant (*lotr2/exo70a1*), displaying a fully delocalized Casparian strip membrane domain and an absence of Casparian strips (CS)^20^. When analyzed at the ultrastructural level, we found a high accumulation of large, vesicle-containing membrane bodies fused with the PM, exclusively in endodermal cells (Fig. 1a). Such bodies were not observed in wild-type at the same stage of differentiation (Fig. 1b,c). We initially thought of this phenotype as a direct consequence of a defective exocyst action in the *lotr2/exo70a1* mutant. However, when we investigated other, unrelated CS-defective mutants, such as *esb1* (*enhanced suberin 1*) or *casp1_casp3* (*casparian strip membrane domain protein 1* and *3*), we found that they equally displayed many such large PM-contiguous bodies, specifically in endodermal cells (Fig. 1b, c, Supplementary Fig. 1a). This indicated that the enhanced presence of these bodies is a response to a defective CS and not a direct consequence of a defective exocyst in the mutant *lotr2/exo70a1*. A 3D reconstruction using FIB-SEM in *lotr2/exo70a1* illustrates the high number and broad distribution of these bodies in a endodermal cell of this mutant (Fig. 1d, Supplementary Fig. 1b, c, Supplementary Movie 1). These large bodies containing extracellular membranes (EVB, Extracellular Vesiculo-tubular membrane-containing Bodies) were between 300 and 900 nm in size (Supplementary Fig. 1d) and their internal structures (extracellular vesicles or tubules) were found to be of varying density (Fig. 1a,c, Supplementary Fig. 1a,e) and between 10 and 100 nm in diameter on 2D sections (Supplementary Fig. 1f).

### Extracellular membranes appear as highly reticulate tubules in tomographic reconstructions

**Fig. 2:**
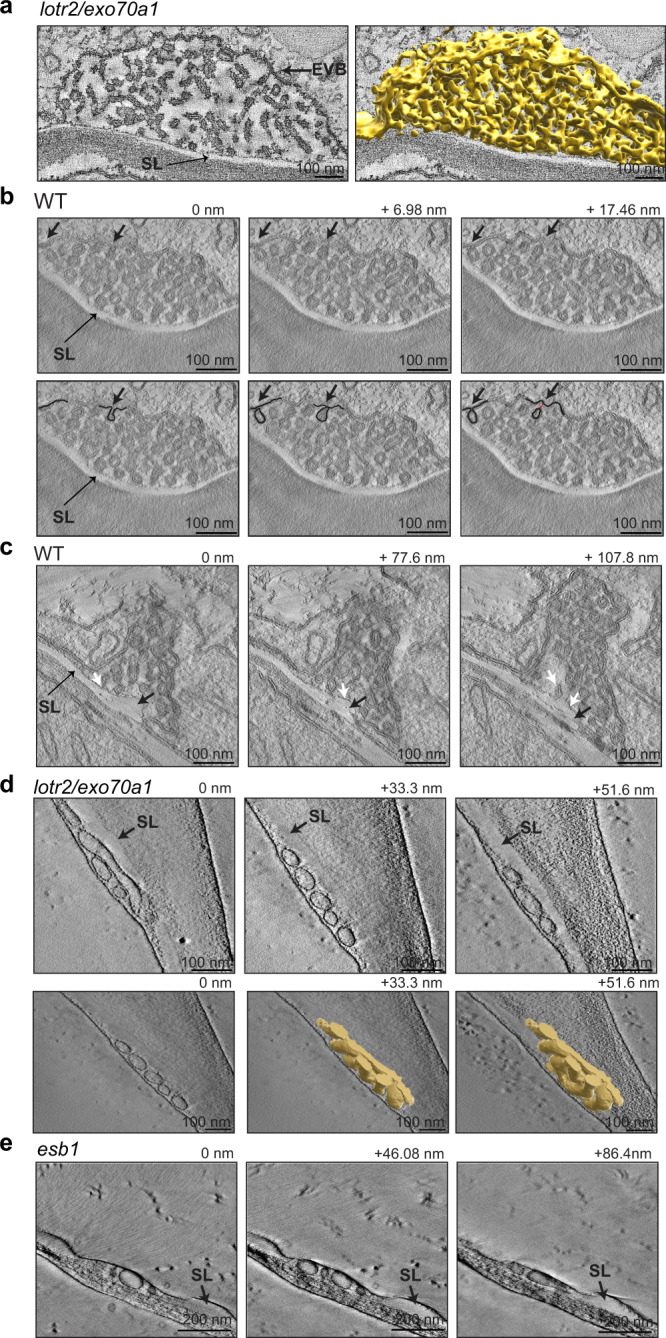
EVB internal organization in chemically and cryo-fixed samples. **a**–**c** Chemically-fixed samples. **d**–**e** Cryo-fixed samples. **a** Single optical tomography slice and 3D reconstruction of one EVB and its inter-connected vesiculo-tubular membranes (segmented in yellow) in *lotr2*/*exo70a1* mutant at 2 mm from tip (see also Supplementary Movie 2). 3D reconstruction performed for 1 representative EVB out of 11 tomograms from 3 individual root sections. **b** Series of three optical sections from a tomogram of one EVB in a WT in the suberizing zone. Arrows highlight the invagination of one vesicle (see also Supplementary Movie 3). Lower panels highlight two invagination events (dark lines) from upper panels (red line highlight a connection between a vesicle and the plasma membrane). **c** Series of three optical sections from a tomogram of one EVB in a WT in the suberizing zone. Black arrows highlight the growing suberin lamellae, white arrows highlight connections between vesiculo-tubular membranes and the suberin lamellae and the small electron dense deposit at the surface of the suberin lamellae that represents the rest of the membrane after fusion (see also Supplementary Movie 4). **b**, **c** Representative pictures for 1 EVB out of 12 tomograms from 4 individual root sections. **d** Tomogram of one EVB in a *lotr2/exo70a1* mutant at 2 mm from root tip. Upper panels show a series of three optical sections, lower panels show the 3D reconstruction of its interconnected vesiculo-tubular membranes (segmented in yellow). Arrows highlight suberin lamellae (SL) (see also Movies S5–S7). 3D reconstruction performed for 1 representative EVB out of 15 tomograms for 3 roots. **e** Series of three optical sections from a tomogram of one EVB in *esb1* mutant at 2 mm from root tip. Arrows highlight suberin lamellae (SL) (see also Supplementary Movies 8–9). Representative EVB out of 14 tomograms from 3 individual root sections.

We undertook tilt-series tomographic reconstructions of single bodies in *lotr2/exo70a1*, as well as in wild-type (WT) root (where these bodies occur later during endodermal development, see below), in order to understand whether the internal structures observed on sections (2D) were indeed vesicles, or rather transversally sectioned tubules. The tomograms suggested that the extracellular membrane structures were part of a highly branched network of tubules (Fig. 2a; Supplementary Movie 2), with little occurrence of isolated vesicles. Close inspection of a number of tomograms allowed us to identify rare cases in which the outer membrane appeared continuous with an inner tubule, suggestive of an EVB generation by active evagination (Fig. 2b; Supplementary Movie 3). We also repeatedly observed a very thin electron-dense layer at the surface of the suberin lamellae in the tomograms (Fig. 2c; Supplementary Movie 4). We then wondered if the classical chemical fixation protocol used in our TEM analysis could have affected the occurrence, shape or internal organization of EVBs. To this end we performed TEM analysis after High Pressure Freezing fixation (HPF, also called cryofixation). After HPF fixation and freeze substitution, we could again observe the presence of extracellular membrane structures in *lotr2/exo70a1* and *esb1* (Fig. 2d, e, Supplemental Fig. 1g). As expected for cryofixed samples, no shrinking of the protoplast and detachment of PM from the cell wall was observed in these samples and the extracellular membrane structures appeared much flatter, lens-shaped. In tilt-series tomographic 3D reconstructions of single EVBs after HPF, we found that the extracellular membranes, although still tubular, appeared larger and more homogenous in size, with less branching, resembling a ginger root in structure and proportion (Fig. 2d, e, Supplementary Movies 5-9). Thus, chemical fixation appears to cause enlargement and consequent invagination of EVBs into the cell, possibly also causing the extracellular membrane tubules to fracture and reticulate, explaining their highly heterogenous appearance. HPF allowed us to confirm the occurrence of EVBs in CS mutants and their absence in WT. It also provided us with a much superior description of their morphology. Yet, our current HPF sample throughput is low, especially due to the fact that the majority of root samples in the differentiated root regions that are of interest to us, are broken. This is due to the presence of large vacuoles and their high-water content making them particularly difficult to freeze without creating ice crystals. This currently does not allow us to obtain sufficient sample sizes and entire root sections required for quantification. We therefore continued to use chemical fixation for further quantitative analysis.

### EVBs are associated with suberization

**Fig. 3:**
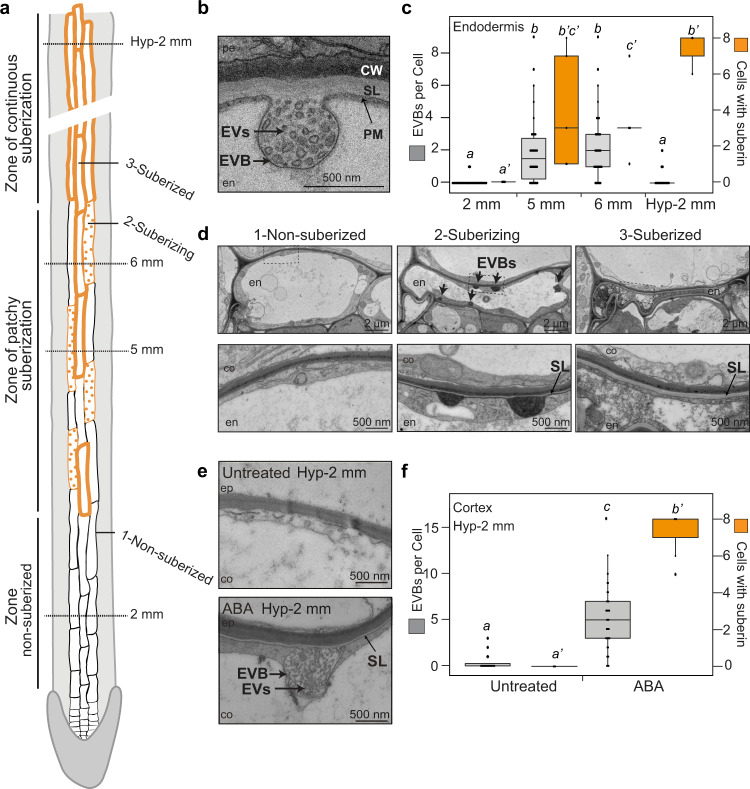
Extracellular vesiculo-tubular membranes accumulate in suberizing cells. **a** Schematic view of suberin differentiation stages in roots. Positions along the roots are marked (2 mm, 5 mm and 6 mm correspond to positions from the root tip and Hyp-2 mm corresponds to position from the hypocotyl-root junction). Examples of non-suberized (1), suberizing (2) and suberized (3) endodermal cells are highlighted. **b**, **d**, **e** TEM sections showing suberin lamellae (**SL**), **EVBs** and **EVs**. **en**, endodermis; **co**, cortex; **pe**, pericycle. **b** Endodermal section in WT at 5 mm from root tip. **c**, **f** Number of visible extracellular vesicular-tubules containing bodies (EVBs), (in grey, left axis) and number of suberized cells (in orange, right axis) in endodermal or cortical layers in TEM cross-sections of full roots. Data are presented as dot plots with box plots overlaid (*n* = 49, 42, 42, 24, 40, 56 cells for EVB number, *n* = 6, 5, 5, 3, 5, 7 root sections for number of suberized cells per layer at in the endodermis at 2, 5, 6 mm and Hyp-2 mm and in the cortex untreated or treated with ABA respectively). For boxplots, box shows the quartiles, whiskers indicate the minimum and maximum non-outlier values, dots located outside the whiskers of the box plots indicate outliers falling outside 1.5 times the interquartile range above the upper quartile and below the lower quartile, and center line corresponds to the median. Different letters indicate significant differences between genotypes or growth conditions (*P* < 0.05). **c** Quantifications in the endodermal layer, in WT plants at different positions. **d** Pictures illustrating the 3 stages of non-suberized, suberizing and suberized endodermal cells from a root section in the zone of patchy suberization in WT plants (see also Supplementary Fig. 2a). Representative pictures from 1 root section out of at least 4 root section in WT in the zone of patchy suberization. **e** Cortical sections in WT plants treated or not with ABA at Hyp-2 mm (see also Supplementary Fig. 2d). **f** Quantifications for the cortical layer at Hyp-2 mm in WT plants treated or untreated with ABA.

The mutants *lotr2/exo70a1*, *esb1* and *casp1_casp3* all affect CS formation in very different ways. One common feature, however, is that they all display enhanced suberin formation closer to the root tip, where it never occurs in wild-type (Fig. 1b)^20–22^. Indeed, in a number of cases, we could observe a striking association between partially formed suberin lamellae and EVBs (Figs. 1a and 2c,e). We therefore investigated if the accumulation of these vesicles was associated with suberin formation in wild-type. Suberin development has been well-described in Arabidopsis roots^23–25^, with a non-suberized zone (at 2 mm), followed by a patchy zone of ongoing suberization (between 4 and 7 mm), and a fully suberized zone (after 7 mm), where all eight endodermal cells in a section are suberized (Fig. 3a). Ultrastructural analysis along this developmental gradient revealed a transient, high accumulation of EVBs associated with the endodermal PM in the patchy, suberizing zone (at 5 and 6 mm, Fig. 3b, c), while their number was neglectable prior to suberin formation, as well as in the fully suberized zone. A tight correlation with suberin formation can also be observed in a single root section in the patchy zone, making use of the fact that in this zone, 3 developmental stages of endodermal cells can be simultaneously observed. i.e., non-suberized, suberizing and suberized (categorized by the presence, thickness and continuity of suberin lamellae in these cells). Here, we only observed a high number of EVBs in the suberizing cells (Fig. 3d, Supplementary Fig. 2a). The dimensions of the EVBs observed in wild-type, as well as the content and size of their vesiculo-tubular structures in sections, were similar to the EVBs observed in *lotr2/exo70a1*, *esb1* and *casp1_casp3* (Supplementary Fig. 2b, c). In order to further strengthen the association between EVBs and suberization, we decided to induce suberin outside of the endodermis by using 1 μM of abscisic acid (ABA) for 14 h, which has previously been described to cause suberin accumulation in cortical cells^24^. Indeed, we observed induction of EVBs in the cortex of ABA-treated plants (Fig. 3e, f, Supplementary Fig. 2d). Again, these EVBs had dimensions, EV content and size matching those observed in the endodermis of CS mutants and suberizing WT endodermal cells (Supplementary Fig. 2b, c). The enhanced suberin deposition of the many CS-defective endodermis mutants is due to stimulation of the SCHENGEN signaling pathway^22,26,27^. Consequently, the *schengen3* (*sgn3*, also called *gassho1*, *gso1*) receptor mutant does not display enhanced suberin formation and is epistatic to *esb1* and *casp1_casp3* (Supplementary Fig. 3a, b). We therefore tested whether, EVB formation in the early differentiating endodermis of CS mutants was also suppressed in *sgn3_esb1* and *sgn3_casp1_casp3* mutants. Indeed, neither *sgn3* nor *sgn3_esb1* and *sgn3_casp1_casp3* mutants displayed EVBs in the early differentiated endodermis (Supplementary Fig. 3a, b). Thus, the strict correlation between EVB presence and suberin formation holds up even when challenged by a second stimulation of suberization, independent from ABA^28^- in this case, peptide receptor-mediated. Together, our data suggests a causal relationship between EVBs and suberin deposition.

### Secretion dependent suberin deposition

**Fig. 4:**
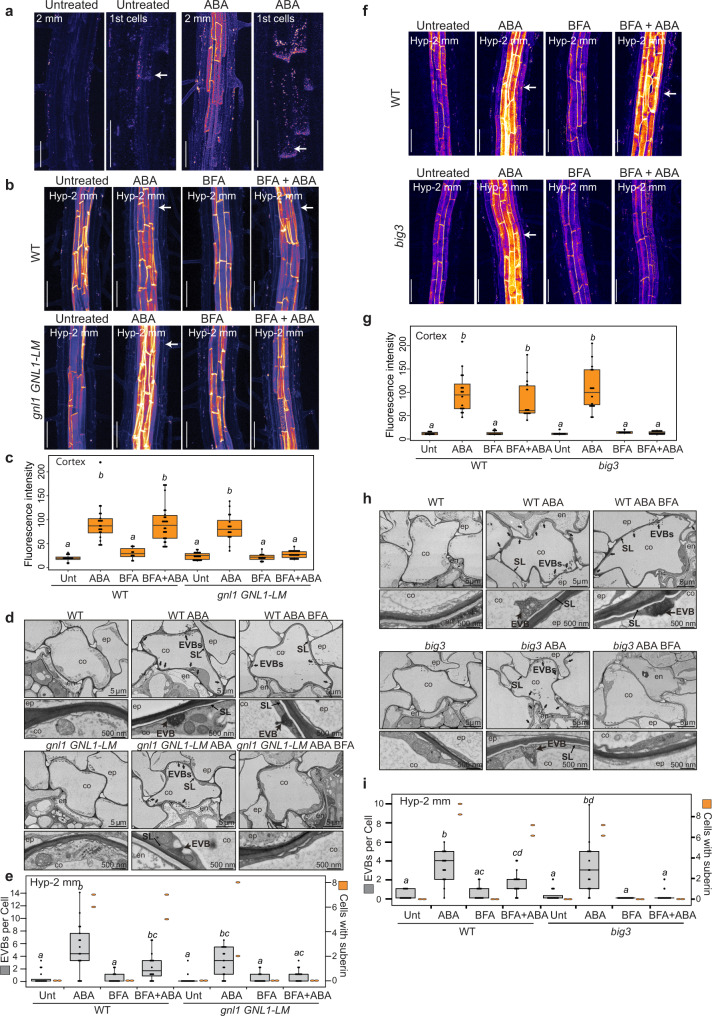
Secretion-dependent suberin deposition. **a,b,f** Fluorol yellow staining for suberin in roots. Fluorescence is presented as Look Up Table (LUT, Fire), scale bars, 50 μm. Representative pictures from at least 10 independent experiments with at least 6 individual roots. **a** WT plants treated or not with ABA. For each condition, left pictures taken at 2 mm from root tip, right pictures display the first suberizing cells (1^st^ cells, highlighted with arrows). **b**, **c** WT and *gnl1GNL1-LM* lines treated or not with ABA and/or BFA. **b** Pictures taken at Hyp-2 mm from hypocotyl. Arrows highlight the cortical suberin. **c** Quantification of maximum fluorescence intensity in cortical-epidermal walls, data presented as box plots (*n* = 14, 22, 10, 28, 22, 16, 14, 28 measures, from 7, 11, 5, 14, 11, 8, 7, 14 individual roots in WT (Unt, ABA, BFA, BFA+ABA) and *gnl1GNL1-LM* (Unt, ABA, BFA, BFA+ABA) respectively), two measures taken per root from opposite cortical cell files, different letters indicate significant differences between genotypes and growth conditions (*P* < 0.05). **d**, **h** TEM sections showing a cortical cell in WT and *gnl1GNL1-LM* lines (**d**) or *big3* mutant (**h**) treated or not with ABA and/or BFA at Hyp-2mm. Arrows highlight EVBs. Lower panels correspond to a magnification from upper panels (zone defined with dashed lines). **e**, **i** Number of EVBs (in grey, left axis) and number of suberized cells (in orange, right axis) in cortical layers TEM cross-sections of full roots in WT and *gnl1GNL1-LM* lines (**e**) and in WT and *big3* mutant (**i**) treated or not with ABA and/or BFA. Data are presented as dot plots with box plots overlaid (*n* = 16, 17, 16, 16, 16, 17, 16, 16, 17, 17, 16, 17, 16, 16, 17, 16 cortical cells for EVB number in WT (Unt, ABA, BFA, BFA+ABA), *gnl1GNL1-LM* (Unt, ABA, BFA, BFA+ABA), WT (Unt, ABA, BFA, BFA+ABA) and *big3* (Unt, ABA, BFA, BFA+ABA) respectively; suberized cells per section were counted for two roots). Different letters indicate significant differences between genotypes and growth conditions for EVB counting (*P* < 0.05). **f**, **g** WT and *big3* mutant treated or not with ABA and/or BFA. **f** Pictures taken at Hyp-2 mm from hypocotyl. Arrows highlight the cortical suberin. **g** Quantification of maximum fluorescence intensity in cortical-epidermal walls, data presented as box plots (*n* = 10, 16, 12, 14, 10, 16, 10, 20 measures, from 5, 8, 12, 14, 10, 16, 10, 20 individual roots in WT (Unt, ABA, BFA, BFA+ABA) and *big3* (Unt, ABA, BFA, BFA+ABA) respectively), two measures taken per root from opposite cortical cell files, different letters indicate significant differences between genotypes or growth conditions (*P* < 0.05). **c**, **e**, **g**, **i** For boxplots, box shows the quartiles, whiskers indicate the minimum and maximum non outlier values, dots located outside the whiskers of the box plots indicate outliers falling outside 1.5 times the interquartile range above the upper quartile and bellow the lower quartile, and center line corresponds to the median.

Suberin is a polyester that is formed as a secondary cell wall, deposited in the form of lamellae just outside of the plasma membrane. Its monomeric precursors are thought to be produced at the endoplasmic reticulum and to be polymerized in the apoplast^18,29^. However, the transport of hydrophobic suberin monomers to the apoplast is poorly understood. Current research focuses mainly on the role of ATP-binding cassette (ABC) transporters and lipid transfer proteins (LTPs), but their functional significance for suberin deposition remains to be demonstrated^18,30,31^. Alternatively, a key role for secretion through vesicles for suberin export has repeatedly been hypothesized, taking into account their hydrophobic nature^18,29^. However, little evidence has been provided for this and earlier observations of EVB-like vesicles in differentiating endodermal and exodermal cells^32,33^, have never entered the literature as evidence for secretion-based suberization^18,29^. We therefore wondered if the vesicles accumulating in suberizing cells reflect suberin monomer secretion to the apoplast. The lipidic nature of suberin allows its staining in whole-mount roots with Fluorol yellow^23–25^. A close look at Fluorol yellow staining in the first suberizing cells of untreated or ABA-treated roots showed a signal not only at the cell periphery (apoplast) but also as punctate structures (Fig. 4a). These structures are smaller than 1 μm and could therefore correspond to the large vesicles observed at the ultrastructural level in suberizing cells. However, Fluorol yellow staining uses harsh conditions and observed subcellular structures cannot be straightforwardly compared with live cell structures or combined with ultrastructural analysis. Nevertheless, since apoplastic suberin, even in the early stages of suberization, occurs exclusively in lamellae, we reasoned that the presence of punctate structures, stained by Fluorol yellow in early suberizing cells, lends some support to the notion of a vesicular, lipidic cargo intermediate during suberization.

In order to study the role of secretory endomembrane trafficking in suberin deposition, we thought to make use of the fact that ABA induces *de novo* suberin formation in cortical cells^24^, allowing us to compare the same cell type in an induced and uninduced state. To address the origin and identity of the EVBs, we screened the Wave Line collection of subcellular markers^34^ upon ABA treatment, but failed to observe cortex-specific changes in fluorescence in any of these lines after treatment (Supplementary Fig. 3c). This was surprising, since the marker collection covers the major intracellular membrane compartments, such as Golgi, trans-Golgi network (TGN), recycling endosomes, vacuoles and MVBs (Supplementary Fig. 3c). We then attempted to affect endomembrane trafficking and secretion and study its consequences on suberization. We refrained from using constitutive trafficking mutants, since they are either weak or have such severe pleiotropic defects that they are not able to undergo proper embryogenesis and root development (e.g.,^35–37^). Since suberization is highly responsive to many different stresses, we predicted that it would be impossible to separate primary from secondary effects in these mutants. We therefore focused on pharmacological interference in order to allow for a more acute manipulation of membrane trafficking in WT and conditional mutants. Brefeldin A (BFA), is a well-characterized inhibitor of membrane trafficking whose mechanism of action on GDP/GTP exchange factors (GEFs) for ARF (ADP-Ribosylation Factor) G-proteins is understood^38^. Importantly, single point mutations can predictably render different ARF-GEFs, acting at different points of the trafficking pathway, either resistant or sensitive to BFA. This effectively allows to use the same inhibitor to be largely specific to endosomal trafficking, to affect trafficking already at the ER-to-cis-Golgi step, or to affect trafficking late in the secretory pathway, at the level of the TGN. This can be achieved by choosing the appropriate genetic background^39–41^. We first used BFA on WT, in combination with ABA treatment, allowing us to observe induced, *de novo* suberin formation in cortical cells. BFA treatment in WT did not affect ABA-induced cortical suberization, nor did it decrease the quantity of EVB structures, suggesting that endosomal trafficking is not required (Fig. 4b–e). However, in *gnl1GNL1-LM* (*gnom-like1*) plants, a genotype with root development indistinguishable from wild-type^40^, ABA-dependent suberin deposition in cortical cell walls was blocked upon BFA treatment (Fig. 4b, c). Importantly, BFA treatment also abrogated the increase of EVBs in cortical cells, induced by ABA (Fig. 4d, e). *gnl1GNL1-LM* is hypersensitive to Brefeldin A (BFA), because, in addition to GNOM, GNL1 is also rendered BFA-sensitive in this background, thus blocking secretory trafficking already at the ER-to-Golgi step^40,42^. We then performed the same analysis with *big3* (*BFA-inhibited-guanine-nucleotide-exchange-factor*) mutant. BIG3 is fully redundant with its homolog BIG1,2 and 4, which all act together at the TGN, and its mutant is indistinguishable from WT. However, BIG3 is the only BFA-resistant ARF-GEF at the TGN. As a consequence, *big3* mutants leads to BFA hypersensitivity, because of an inhibition of all TGN-localized BIGs, causing a block in late secretion from the TGN to the PM^43^. As observed in *gnl1GNL1-LM*, BFA treatment blocked ABA-induced cortical suberization in the mutant *big3* (Fig. 4f, g). Moreover, in *big3* mutant, BFA treatment also impaired the increase of EVBs in cortical cells after ABA treatment (Fig. 4h, i).

Altogether these pharmacogenetic approaches indicate that secretory endomembrane trafficking is required for suberin deposition in the cell wall, whereas affecting exclusively endosomal trafficking with BFA appears to be ineffective to block EVB formation and suberin deposition.

### Proposed models for suberin monomer transport by vesiculo-tubular intermediates

**Fig. 5:**
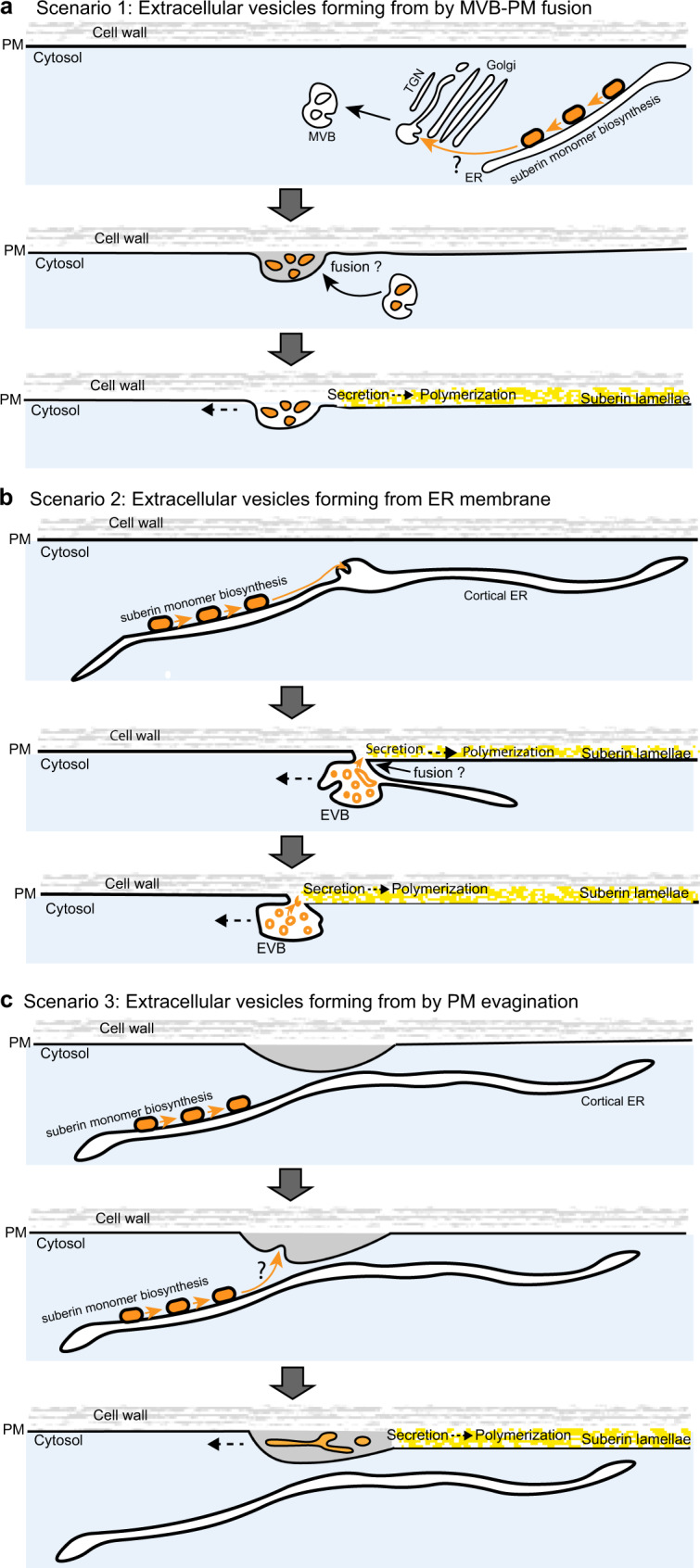
Speculative models for suberin monomer transport by vesiculo-tubular intermediates. **a** In scenario 1, suberin monomers, produced at the endoplasmic reticulum (ER) might be transported, directly, or via Golgi cisternae and trans-Golgi network (TGN), to multi-vesicular bodies (MVBs), where they would accumulate in intra-luminal vesicles. Fusion of MVBs with the PM would place suberin monomer-containing vesicle into the apoplast. **b** In scenario 2 the lipid-like suberin monomers, produced by the successive activities of their biosynthetic enzymes (orange blocks) at the endoplasmic reticulum (ER) might associate into cortical ER-derived subdomains (orange) that evaginate into the lumen of the ER. The initially ER-derived structure swells, accommodating larger amounts of monomer-containing tubules (orange) and eventually disconnects from the ER and fuses with the nearby plasma membrane. Eventually, the suberin monomer containing tubules are placed in the apoplast and are gradually consumed as substrates of cell wall localized suberin polymerizing enzymes, forming suberin lamellae (yellow). **c** In scenario 3, suberin monomers, produced at the endoplasmic reticulum (ER) might associate with the inner face of the plasma membrane, which, upon evagination and scission, would place monomer-containing tubules (orange) into the apoplast.

It has been proposed previously for the process of EV biogenesis that these structures might stem from the MVB/LE pathway^1,6^, with MVBs being re-directed for fusion with the PM, which would place their intra-luminal vesicles in the extracellular space. Support for this model comes from the overall structural and topological resemblance of EVBs and MVBs (see scenario 1 in Fig. 5a). Yet, the structural resemblance of EVBs with MVBs largely disappears in cryofixed samples and we were unable to observe a comparable presence of MVBs, as we see for EVBs in suberizing cells. Moreover, the massive increase in EVBs upon ABA treatment in cortical cells is not reflected in observable changes in MVB/LE numbers or size, when using our Wave marker lines (Supplementary Fig. 3c). Lastly, interfering with endosomal trafficking by BFA treatment in wild-type, did not affect EVB formation or suberization (Fig. 4b–i). By contrast, inhibiting early and late secretory trafficking at the level of the ER by BFA treatment of the *gnl1GNL1-LM* mutant or TGN by BFA treatment of the *big3* mutant did affect EVB formation and suberization (Fig. 4b–i).

Direct transfer of lipids between ER and PM is known to occur at ER-PM contact sites and to be crucial for cellular membrane homeostasis^44,45^. It could thus be hypothesized that the lipid-like suberin precursors, which are synthesized at the ER, could be transferred directly between ER and PM (see scenario 2 in Fig. 5b). Similar to lipid droplets, shown to burgeon from the ER^46^, luminal tubules rich in suberin monomer lipids might initially form at their site of biogenesis in the ER and then fuse with the PM, placing suberin monomer-containing tubules into the apoplast where they can be polymerized (Fig. 5b). Intriguingly, lipid droplet formation also involves GPATs (GLYCEROL-3-PHOSPHATE SN-2-ACYLTRANSFERASES), the same enzyme class that is catalyzing the last step(s) of suberin/cutin monomer biosynthesis^47^. In addition, lipid droplet homeostasis was shown to depend on the ARF1/COPI (ARF1/COAT PROTEIN 1) machinery^47^, which is precisely the step sensitized to BFA action in the *gnl1GNL1-LM* background. However, we are not aware of examples of direct fusions of ER subdomains with the PM, which is required in such a scenario. Also, we could not observe any cases of dilated ER domains, or instances of ER-PM fusions in our mutants or ABA-stimulated samples.

Lastly, extracellular membrane tubules could form through direct evagination of plasma membrane into the cell wall (see scenario 3 in Fig. 5c). Indeed, our HPF protocol happened to stain only PM and extracellular tubules, suggestive of a similar lipid composition of both membranes. This simple scenario of tubule formation, however, does not explain how suberin monomers would be placed inside those tubules and a transport of monomers from the ER membrane to the evaginating PM would have to be postulated.

Whatever the precise mechanism of EV biogenesis, it is important to consider the nature and possible function of EVs in suberization. We currently presume that these extracellular vesiculo-tubular structures could carry the lipid-like, suberin monomeric precursors either in the membranes themselves, or within the lumen of the tubules. In both cases, fusion with the hydrophobic surface of the suberin lamella could release these monomers and make them available to the apoplastic suberin polymerizing enzymes. Indeed, we often observe a tight apposition of EVs with the growing ledge of suberin lamellae and in some cases could observe structures resembling fusion intermediates or remnant membranes on the surface of the lamellae (Fig. 2c). Yet, in light of the broad presence of EVBs in many aspects of plant biology, it has to be pointed out that, despite the intimate association we observe between EVBs and suberin lamellae formation, EVBs might play a more general role in cell wall formation. This could be some process that would strictly co-occur and be required for suberization, but that do not involve the actual transport of suberin monomeric precursors. We have currently no way of definitively addressing this point, but we believe that the highly inducible and reproducible process of EVB formation that we describe here is a promising model to investigate the elusive role of EVBs in plant cell wall formation and plant biology in general.

Finally, our model of extracellular tubule-mediated suberin monomer transport generates a tantalizing parallel to the extracellular tubulo-vesicular structures observed in arbuscules at the fungal plant membrane interface^8,9^. There is good evidence that lipid-like molecules are provided to the fatty acid-auxotrophic fungus and that their generation requires GPAT enzymes^48^. We therefore speculate that there might be a deep cellular and molecular resemblance of the mechanisms providing lipidic molecules to the fungus at the arbuscule and the process of transporting very similar, lipid-like suberin monomers to the apoplast during suberization. Our work here establishes a developmental role for EVBs in the formation of suberin, a major cell wall polymer, distinct from the more widely reported functions of EVBs in symbiotic and pathogenic interactions.

## Methods

### Material

All experiments were performed with Arabidopsis, ecotype Columbia. Mutants and transgenic lines analyzed in this study were generated and characterized before: *exo70a1-4/lotr2-1*^20^, *sgn3-3*^22^*, esb1-1*^21^*, casp1_casp3*^49^*, sgn3-3_esb1-1*^22^*, sgn3-3_casp1_casp3*^22^*, gnl1-1GNL1-LM*^40^*, big3*^43^ and the following Wave lines^34^ Wave2Y (*UBQ10::RabF2b/ARA7-EYFP*), Wave3Y (*UBQ10::RabC1-EYFP)*, Wave5Y (*UBQ10::RabG3f-EYFP)*, Wave7Y (*UBQ10::RabF2a/Rha1-EYFP)* and Wave13Y (*UBQ10::VTI12-EYFP)*. The corresponding gene numbers are as follows: *BIG3*, At1g01960; *CASP1*, At2g36100; *CASP3*, At2g27370; *ESB1*, At2g28670; *EXO70A1/LOTR2*, At5g03540; *GNL1*, At5g39500; *GPAT5*, At3g11430; *SGN3*, At4g20140.

### Growth conditions

For all experiments, seeds were surface sterilized, sown on 0.5 x MS (Murashige and Skoog) 0.8% agar plates, incubated 2 to 3 days at 4 °C and grown vertically in growth chambers at 22 °C, under continuous light (100 μE). All analyses were performed on 5-day-old seedlings. Treatments were performed as transfers for 16 h in a way that seedlings were 5-day-old at the point of analysis. ABA and BFA were directly added to 0.5 x MS plates at the following concentrations: 1 μM and 25 μM, respectively, for 14 h.

### Classical TEM analysis

Plants were fixed in glutaraldehyde solution (EMS, Hatfield, PA) 2.5% in phosphate buffer (PB 0.1 M [pH 7.4]) for 1 h at RT and postfixed in a fresh mixture of osmium tetroxide 1% (EMS, Hatfield, PA) with 1.5% of potassium ferrocyanide (Sigma, St. Louis, MO) in PB buffer for 1 h at RT. The samples were then washed twice in distilled water and dehydrated in ethanol solution (Sigma, St Louis, MO, US) at graded concentrations (30%–40 min; 50%–40 min; 70%–40 min; 100%–2x 1 h). This was followed by infiltration in Spurr resin (EMS, Hatfield, PA, US) at graded concentrations (Spurr 33% in ethanol - 4 h; Spurr 66% in ethanol - 4 h; Spurr 100%–2x 8 h) and finally polymerized for 48 h at 60 °C in an oven. Ultrathin sections of 50 nm thickness were cut transversally at 2, 5, and 6 mm from the root tip and at 2 mm below the hypocotyl-root junction, using a Leica Ultracut (Leica Mikrosysteme GmbH, Vienna, Austria), picked up on a copper slot grid 2 x 1 mm (EMS, Hatfield, PA, US) coated with a polystyrene film (Sigma, St Louis, MO, US). Sections were post-stained with uranyl acetate (Sigma, St Louis, MO, US) 4% in H_2_O for 10 min, rinsed several times with H_2_O followed by Reynolds lead citrate in H_2_O (Sigma, St Louis, MO, US) for 10 min and rinsed several times with H_2_O. Micrographs were taken with a transmission electron microscope Philips CM100 (Thermo Fisher Scientific, Waltham, MA USA) at an acceleration voltage of 80 kV with a TVIPS TemCamF416 digital camera (TVIPS GmbH, Gauting, Germany) using the software EM-MENU 4.0 (TVIPS GmbH, Gauting, Germany). Panoramic alignments were performed with the software IMOD^50^.

### High Pressure freezing and cryo-substitution

For the High Pressure Freezing, pieces of root 5 mm long were cut from tip, and then placed in an aluminum planchet of 6 mm in diameter with a cavity of 0.1 mm (Art.610, Wohlwend GmbH, Sennwald, Switzerland) filled with 15% Dextran in 2-morpholinoethanesulfonic acid buffer (MES 50 mM, [pH 5.7]) covered with a tap planchet (Art.611, Wohlwend GmbH, Sennwald, Switzerland) and directly high pressure freezed using a High Pressure Freezing Machine HPF Compact 02 (Wohlwend GmbH, Sennwald, Switzerland). The samples were then chemically fixed, dehydrated and infiltrated with resin at cold temperature using the Leica AFS2 freeze substitution machine (Leica Mikrosysteme GmbH, Vienna, Austria) with the following protocol: Dehydration and fixation in a solution containing a mixture of osmium tetroxide 0.5% (EMS, Hatfield, PA) with glutaraldehyde 0.5% (EMS, Hatfield, PA) with uranyl acetate 0.1% (Sigma, St. Louis, MO) in acetone (Sigma, St Louis, MO, US) at graded temperature (−90 °C for 30 h; from −90 °C to −60 °C in 6 h; −60 °C for 10 h; from −60 °C to −30 °C in 6 h; −30 °C for 10 h; from −30 °C to 0° in 6 h) This was followed by washing in acetone and then infiltration in Spurr resin (EMS, Hatfield, PA, US) at graded concentration and temperature (30% for 10 h from 0 °C to 20 °C; 66% for 10 h at 20 °C; 100% twice for 10 h at 20 °C) and finally polymerized for 48 h at 60 °C in an oven.

### Focused ion beam scanning electron microscopy (FIB-SEM)

The resin block was oriented and mounted on an aluminium support, glued with conductive resin Epotek H20S® (EMS, Hatfield, PA, US), and polymerized overnight in an oven at 60 °C. It was then trimmed in the ultramicrotome to position the sample (2 mm from the root tip) and prepare its geometry for FIB-SEM analysis. 30 nm of platinum was then sputter coated on the block using a Leica EM SCD 500 sputter coater (Leica Mikrosysteme GmbH, *Vienna*, Austria). Serial block face imaging is finally performed in a Helios NanoLab 650 (Thermo Fisher Scientific, Waltham, MA USA), using the FEI Slice and View software™. The milling of 40 nm slice thickness was done at 30 kV acceleration voltage and 6.6 nA current. The cross section images were acquired by detecting backscattered electrons with the In-column detector (ICD) in immersion mode, at 4.2 mm of working distance and an electron beam of 2 kV, 800 pA and 5 μs of dwell time with a frame of 4096 × 3536 pixels, a horizontal field width of 56 µm and a pixel size of 13.6 nm, total Z volume acquired is 27.96 μm. Further details on block geometry and milling strategy were previously described in^51^. Volume alignment and 3D modelling were performed using IMOD software^50^.

### TEM tomography and 3D reconstruction

For electron tomography, semi-thin sections of 250 nm thickness were cut transversally to the root using a Leica Ultracut (Leica Mikrosysteme GmbH, Vienna, Austria) and then, picked up on 75 square mesh copper grids (EMS, Hatfield, PA, US). Sections were post-stained on both sides with uranyl acetate (Sigma, St Louis, MO, US) 2% in H_2_O for 10 min and rinsed several times with H_2_O. Protein A Gold 10 nm beads (Aurion, Wageningen, The Netherlands) were applied as fiducials on both sides of the sections and the grids were placed on a dual axis tomography holder (Model 2040, Fischione Instruments). The area of interest was taken with a transmission electron microscope JEOL JEM-2100Plus (JEOL Ltd., Akishima, Tokyo, Japan) at an acceleration voltage of 200 kV with a TVIPS TemCamXF416 digital camera (TVIPS GmbH, Gauting, Germany) using the SerialEM software package^52^. Micrographs were taken as single or dual-axis tilt series over a range of −60° to +60° using SerialEM at tilt angle increment of 1°. Tomogram reconstruction was done with IMOD software^50^, segmentation with Ilastik software package^53^ and model visualization with Imaris software package (Oxford Instruments).

### Fluorescence microscopy

Fluorol yellow (FY) staining was used to visualize suberin in whole-mounted roots as described before^25^. Seedlings were incubated in freshly prepared FY 088 (0.01% w/v, lactic acid) at 70 °C for 30 min, rinsed with water and counterstained with aniline blue (0.5% w/v, water) at RT for 30 min in darkness, washed, mounted in 70% glycerol and observed with confocal. For visualization of cell files in live imaging, 5-day-old seedlings were incubated in the dark for 10 min in a fresh solution of 15 mM (10 mg/ml) Propidium Iodide (PI) dissolved in liquid 0.5 x MS and rinsed in liquid 0.5 x MS prior to imaging. Confocal laser scanning microscopy experiments were performed on a Zeiss LSM 700 or Zeiss LSM 880. Excitation and detection windows were set as follows: FY 488 nm, SP 640 nm; YFP 488 nm, 500–530 nm; PI 555 nm, SP 640 nm. For FY imaging laser power was reduced as low as 0.2% to limit bleaching. Confocal pictures were subsequently analyzed with Fiji^54^, channels merged, Z stacks converted as 3D-projections and/or orthogonal views. FY staining is presented as Look Up Tables (LUT). Fluorescence in the cortex was quantified from pictures taken with exactly the same parameters, by tracing a 10 μm line crossing the cell wall between epidermal and cortical cells, measuring the fluorescence intensity and considering the maximum value per measure (2 measures taken by root from opposite cortical cell files).

### Statistical analysis

All graphics and statistical analyses were done in the R environment. For multiple comparisons between genotypes or conditions, one-way ANOVA was performed, and Tukey’s test subsequently used as a multiple comparison procedure. When the data did not follow the linear model assumption Kruskal-Wallis and nonparametric Tukey’s test were performed for multiple comparison. Statistical differences (p-values) are presented in Supplementary Table 1.

### Reporting summary

Further information on research design is available in the Nature Research Reporting Summary linked to this article.

## Supplementary information

Supplementary Information

Description of Additional Supplementary Files

Supplementary Movie 1

Supplementary Movie 2

Supplementary Movie 3

Supplementary Movie 4

Supplementary Movie 5

Supplementary Movie 6

Supplementary Movie 7

Supplementary Movie 8

Supplementary Movie 9

Reporting Summary

## Data Availability

All data generated during this study are included in this published article (and its Supplementary Information Files). Source data are provided with this paper.

## Source data

Source Data
