# Supplementary material for: Extracellular vesiculo-tubular structures associated with suberin deposition in plant cell walls
Source: Nat Commun. 2022 Mar 18;13:1489. doi: 10.1038/s41467-022-29110-0 (PMC8933581; doi:10.1038/s41467-022-29110-0)
Supplement: Supplementary file 1 — Supplementary Information [file 41467_2022_29110_MOESM1_ESM.pdf]

Extracellular vesiculo-tubular structures associated with suberin deposition in plant cell walls

De Bellis *et al.*,

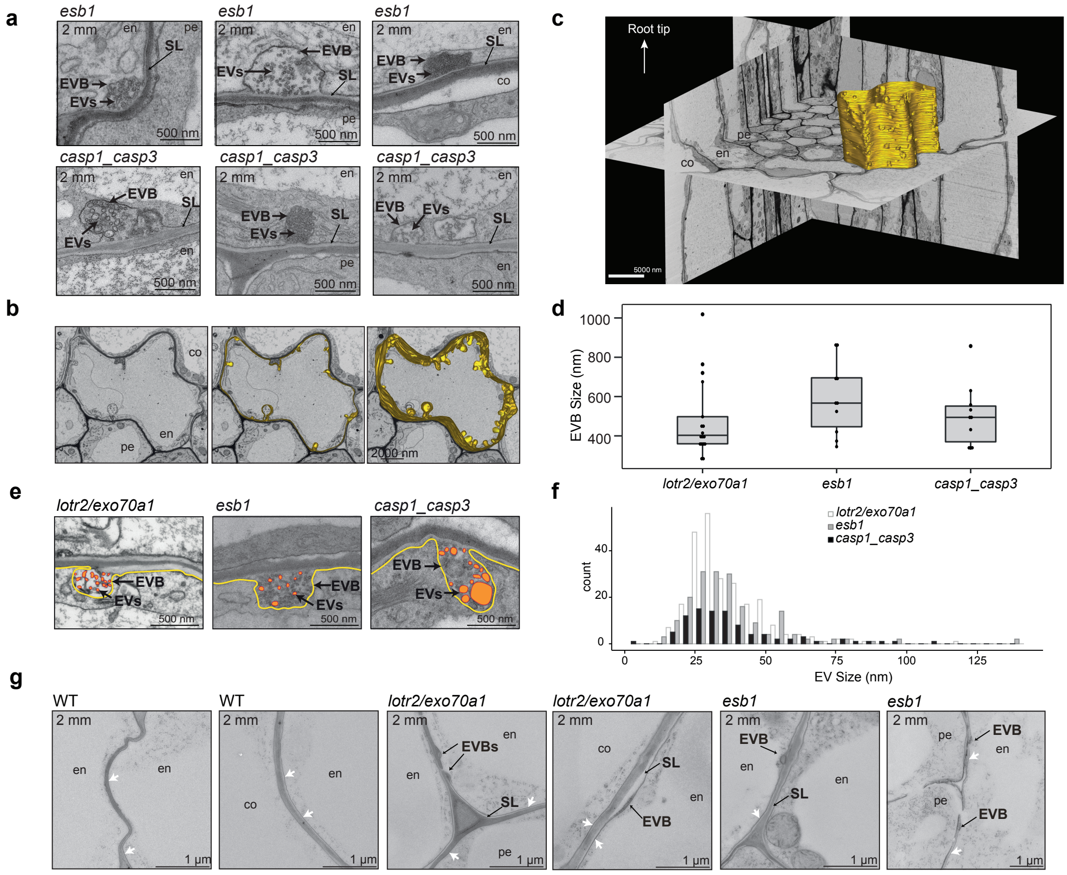

Supplementary Fig. 1. **Extracellular vesicular-tubules accumulate in Casparian strip mutants. a.** TEM sections showing suberin lamellae (SL), extracellular vesicular-tubules (EVs) and EV containing bodies (EVBs) in endodermal (en) cells in *esb1* and *casp1_casp3* at 2 mm from root tip. Arrows highlight EVBs, co, cortex; pe, pericycle. Representative pictures in a portion of 1 endodermal cell out of 8 and 5 individual root sections from 4 and 3 independent experiments for *esb1* and *casp1_casp3* respectively. **b-c.** 3D model of the PM and its EVBs (in yellow) in *lotr2/exo70a1* mutant (see also Fig. 1d and Movie S1). The model was done on a Z portion of 10 µm starting at 2 mm from the root tip (250 sections of 40 nm thickness) from a FIB-SEM stack from 1 root section. **b.** Screen shots from Movie S1. **c.** Orthogonal slices (xy,xz,zy) view with 3D model view of the PM and its EVBs. **d.** Size of EVBs observed in *lotr2/exo70a1, esb1* and *casp1_casp3.* No significant difference was observed after ANOVA analysis. Data are presented as dot plots with box plots overlaid (n=17, 10, 10 EVB from 7, 8, 5 root sections for *lotr2/exo70a1*, *esb1* and *casp1_casp3* respectively). For boxplots, box shows the quartiles, whiskers indicate the minimum and maximum non-outlier values, dots located outside the whiskers of the box plots indicate outliers falling outside 1.5 times the interquartile range above the upper quartile and bellow the lower quartile, and center line corresponds to the median. **e.** Highlight of the PM and its EVBs (highlighted in yellow) and EVs (highlighted in orange) in *lotr2/exo70a1*, *esb1* and *casp1_casp3* mutants (pictures from Fig. 1a and c). **f.** EV size distribution, estimated after quantification from 2D TEM sections in *lotr2/exo70a1, esb1* and *casp1_casp3* (n=245, 184, 92 EV from 7, 8, 5 root sections for *lotr2/exo70a1*, *esb1* and *casp1_casp3* respectively). **g.** TEM sections from cryofixed samples showing the presence or absence of EVBs in endodermal cells at 2 mm from root tip in WT, *lotr2/exo70a1* and *esb1.* White arrows highlight the plasma membrane tightly attached to the cell wall without any plasmolysis. Representative pictures in a portion of 1 endodermal cell out of 3, 5, 5 root sections from 3 independent experiments for in WT, *lotr2/exo70a1* and *esb1* respectively.

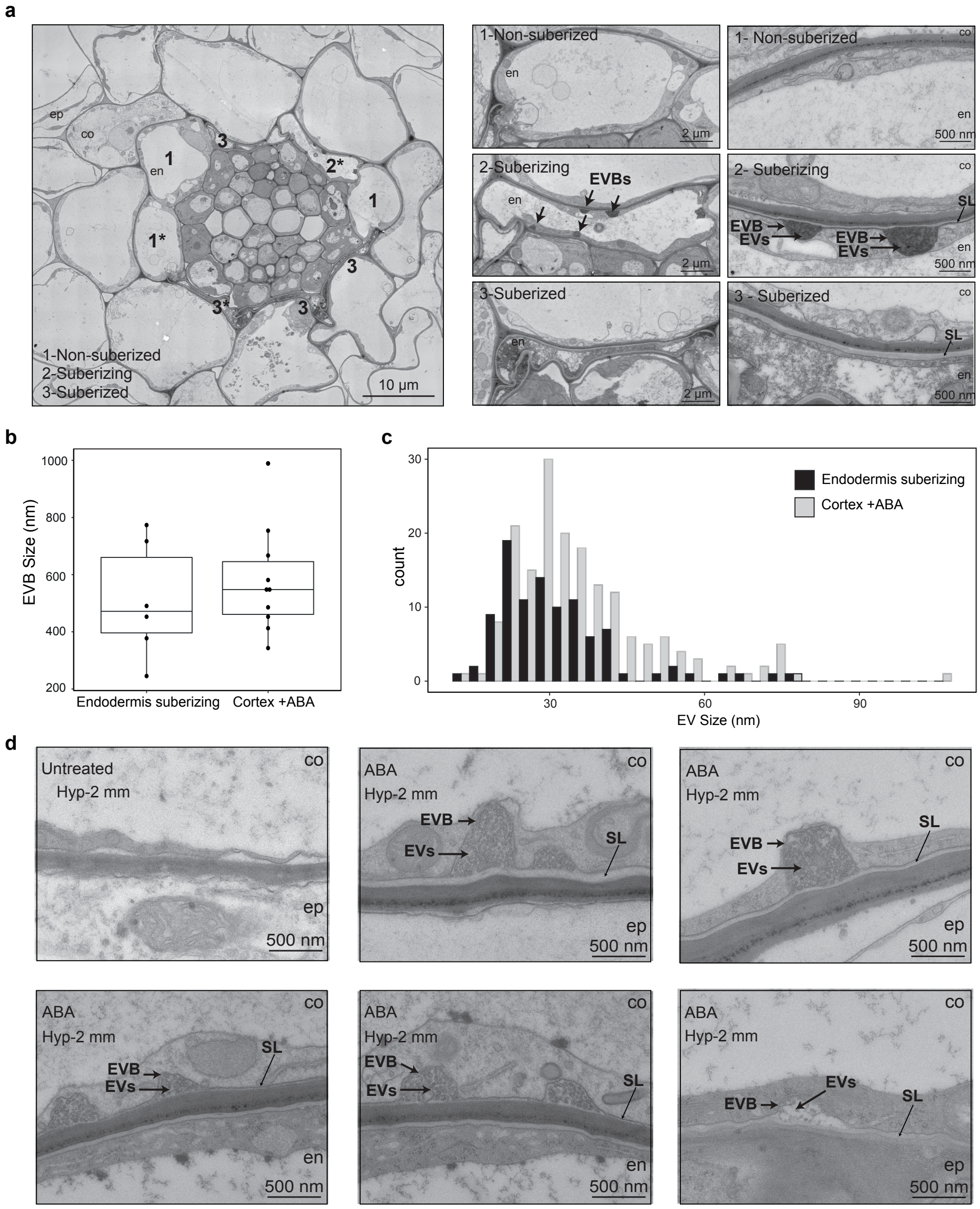

Supplementary Fig. 2. **Extracellular vesicular-tubules accumulate in suberizing cells. a, d.** TEM sections showing suberin lamellae (SL), extracellular vesicular-tubules (EVs) and EV containing bodies (EVBs) in endodermal (en) or cortical cells (co). Arrows highlight EVBs, pe, pericycle. **a.** Section of a WT root in the zone of patchy suberization with the three differentiations stages 1-Non-suberized, 2-Suberizing, 3-Suberized. Asterisk indicates the endodermal cells highlighted in the right panels and in Fig. 3d at different scales. Representative pictures from 1 root section out of at least 4 root section in WT in the zone of patchy suberization. **b.** Size of EVBs observed in WT plants in suberizing endodermal cells and in cortical cells after ABA treatment. No significant difference was observed after ANOVA analysis. Data are presented as dot plots with box plots overlaid (n=6 and 10 EVB from 5 root sections for endodermis suberizing and cortex +ABA respectively). For boxplots, box shows the quartiles, whiskers indicate the minimum and maximum non-outlier values, dots located outside the whiskers of the box plots indicate outliers falling outside 1.5 times the interquartile range above the upper quartile and bellow the lower quartile, and center line corresponds to the median. **c.** EV size distribution, estimated after quantification from 2D TEM sections from WT plants in suberizing endodermal cells and in cortical cells after ABA treatment (n=99 and 175 EV from 5 root sections for endodermis suberizing and cortex +ABA respectively). **d.** Cortical sections in WT roots treated or not with ABA, at 2 mm from hypocotyl-root junction. Representative pictures in the portion of 1 cortical cell out of 9 untreated and 10 ABA treated individual root sections from 3 independent experiment.

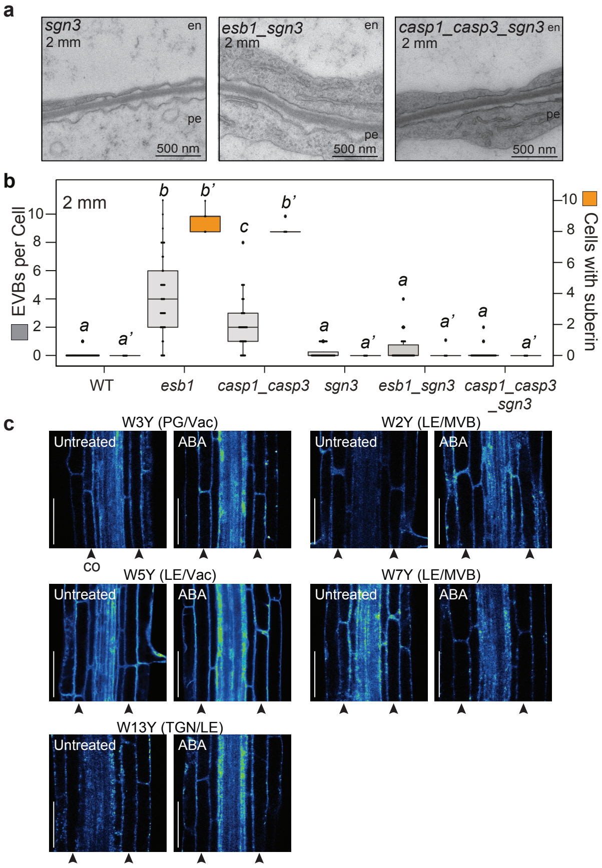

Supplementary Fig. 3. **Secretion-dependent suberin deposition. a.** TEM sections illustrating the absence of suberin lamellae and EVBs in endodermal (en) cells of *sgn3, esb1_sgn3* and *casp1_casp3_sgn3* mutants at 2 mm from root tip. Representative pictures in the portion of 1 endodermal cell out of 5 individual root sections from 1 experiment. **b.** Number of visible EVBs (in grey, left axis) and number of suberized cells (in orange, right axis) in endodermal cells 2mm from root tip in TEM cross-sections of full roots. Data are presented as dot plots with box plots overlaid (n=49, 44, 41, 44, 42, 38 EVBs per cells, n=6, 5, 5, 5, 5, 5 suberized cells per section for WT, *esb1*, *casp1_casp3*, *sgn3*, *esb1_sgn3*, *casp1_casp3_sgn3* respectively). For boxplots, box shows the quartiles, whiskers indicate the minimum and maximum non-outlier values, dots located outside the whiskers of the box plots indicate outliers falling outside 1.5 times the interquartile range above the upper quartile and bellow the lower quartile, and center line corresponds to the median. Different letters indicate significant differences between genotypes or growth conditions ($P < 0.05$). Data for WT, *esb1* and *casp1casp3* are also shown in Fig. 1b. **c.** Observation of Wave line markers for endomembrane compartments (TGN, Trans Golgi Network; LE, Late Endosome; MVB, Multivesicular body; PG, Post Golgi; Vac, Vacuole). Pictures were taken at Hyp-2 mm from hypocotyl-root junction (in the zone where cortical suberin is induced by ABA). Fluorescence is presented as Look Up Table (LUT, GreenFireBlue). Arrows highlight the cortical layer. Scale bars, 50 µm. Representative pictures out of at least 7 individual roots from 2 independent experiments.

Supplementary Table 1. **Statistical differences**

Figure	Samples		Test	DF	p-value
Figure 1b	WT	*lotr2*	Non parametric Tukey	44	0,00E+00
	WT	*esb1*			0,00E+00
	WT	*casp1casp3*			0,00E+00
	lotr2	*esb1*			1,00E+00
	lotr2	*casp1casp3*			4,79E-04
	esb1	*casp1casp3*			1,58E-03
	WT	*lotr2*	Non parametric Tukey	7	0,00E+00
	WT	*esb1*			0,00E+00
	WT	*casp1casp3*			0,00E+00
	lotr2	*esb1*			9,96E-01
	lotr2	*casp1casp3*			9,36E-01
	esb1	*casp1casp3*			6,20E-01
Figure 3c	WT_2mm	WT_5mm	Non parametric Tukey	29	4,99E-10
	WT_2mm	WT_6mm			2,10E-13
	WT_2mm	WT_hypo2mm			5,96E-01
	WT_5mm	WT_6mm			9,72E-01
	WT_5mm	WT_hypo2mm			8,18E-06
	WT_6mm	WT_hypo2mm			1,78E-07
	WT_2mm	WT_5mm	Non parametric Tukey	5	0,00E+00
	WT_2mm	WT_6mm			0,00E+00
	WT_2mm	WT_hypo2mm			0,00E+00
	WT_5mm	WT_6mm			1,00E+00
	WT_5mm	WT_hypo2mm			6,53E-01
	WT_6mm	WT_hypo2mm			4,76E-02
Figure 3f	WT_Cortex_unt	WT_Cortex_ABA	Non parametric Tukey*	29	0,00E+00
	WT_Cortex_unt	WT_Cortex_ABA	Non parametric Tukey*	5	0,00E+00
Figure 4c	WT_unt	WT_ABA	Non parametric Tukey	11	0,00E+00
	WT_unt	WT_BFA			5,23E-01
	WT_unt	WT_ABABFA			0,00E+00
	WT_unt	*gnl1GNLLM*_unt			9,07E-01
	WT_unt	*gnl1GNLLM*_ABA			0,00E+00
	WT_unt	*gnl1GNLLM*_BFA			1,00E+00
	WT_unt	*gnl1GNLLM*_ABABFA			8,58E-02
	WT_ABA	WT_BFA			0,00E+00
	WT_ABA	WT_ABABFA			1,00E+00
	WT_ABA	*gnl1GNLLM*_unt			0,00E+00
	WT_ABA	*gnl1GNLLM*_ABA			1,00E+00
	WT_ABA	*gnl1GNLLM*_BFA			0,00E+00

	WT_ABA	*gnl1GNLLM*_ABABFA			0,00E+00
	WT_BFA	WT_ABABFA			2,02E-11
	WT_BFA	*gnl1GNLLM*_unt			9,70E-01
	WT_BFA	*gnl1GNLLM*_ABA			3,10E-08
	WT_BFA	*gnl1GNLLM*_BFA			7,68E-01
	WT_BFA	*gnl1GNLLM*_ABABFA			1,00E+00
	WT_ABABFA	*gnl1GNLLM*_unt			0,00E+00
	WT_ABABFA	*gnl1GNLLM*_ABA			1,00E+00
	WT_ABABFA	*gnl1GNLLM*_BFA			0,00E+00
	WT_ABABFA	*gnl1GNLLM*_ABABFA			0,00E+00
	*gnl1GNLLM*_unt	*gnl1GNLLM*_ABA			0,00E+00
	*gnl1GNLLM*_unt	*gnl1GNLLM*_BFA			1,00E+00
	*gnl1GNLLM*_unt	*gnl1GNLLM*_ABABFA			9,54E-01
	*gnl1GNLLM*_ABA	*gnl1GNLLM*_BFA			0,00E+00
	*gnl1GNLLM*_ABA	*gnl1GNLLM*_ABABFA			0,00E+00
	*gnl1GNLLM*_BFA	*gnl1GNLLM*_ABABFA			5,75E-01
Figure 4e	WT_unt	WT_ABA	Non parametric Tukey	17	1,38E-03
	WT_unt	WT_BFA			1,00E+00
	WT_unt	WT_ABABFA			4,35E-02
	WT_unt	*gnl1GNLLM*_unt			9,87E-01
	WT_unt	*gnl1GNLLM*_ABA			7,71E-03
	WT_unt	*gnl1GNLLM*_BFA			1,00E+00
	WT_unt	*gnl1GNLLM*_ABABFA			7,22E-01
	WT_ABA	WT_BFA			2,14E-03
	WT_ABA	WT_ABABFA			3,98E-01
	WT_ABA	*gnl1GNLLM*_unt			2,43E-04
	WT_ABA	*gnl1GNLLM*_ABA			8,36E-01
	WT_ABA	*gnl1GNLLM*_BFA			2,25E-03
	WT_ABA	*gnl1GNLLM*_ABABFA			4,06E-02
	WT_BFA	WT_ABABFA			3,82E-02
	WT_BFA	*gnl1GNLLM*_unt			9,33E-01
	WT_BFA	*gnl1GNLLM*_ABA			8,48E-03
	WT_BFA	*gnl1GNLLM*_BFA			1,00E+00
	WT_BFA	*gnl1GNLLM*_ABABFA			7,12E-01
	WT_ABABFA	*gnl1GNLLM*_unt			3,47E-03
	WT_ABABFA	*gnl1GNLLM*_ABA			9,89E-01
	WT_ABABFA	*gnl1GNLLM*_BFA			3,90E-02
	WT_ABABFA	*gnl1GNLLM*_ABABFA			5,59E-01
	*gnl1GNLLM*_unt	*gnl1GNLLM*_ABA			6,98E-04
	*gnl1GNLLM*_unt	*gnl1GNLLM*_BFA			9,33E-01
	*gnl1GNLLM*_unt	*gnl1GNLLM*_ABABFA			1,12E-01

	*gnl1GNLLM*_ABA	*gnl1GNLLM*_BFA			8,53E-03
	*gnl1GNLLM*_ABA	*gnl1GNLLM*_ABABFA			1,65E-01
	*gnl1GNLLM*_BFA	*gnl1GNLLM*_ABABFA			7,09E-01
Figure 4g	WT_unt	WT_ABA	Non parametric Tukey	14	0,00E+00
	WT_unt	WT_BFA			1,00E+00
	WT_unt	WT_ABABFA			0,00E+00
	WT_unt	*big3*_unt			1,00E+00
	WT_unt	*big3*_ABA			0,00E+00
	WT_unt	*big3*_BFA			1,00E+00
	WT_unt	*big3*_ABABFA			1,00E+00
	WT_ABA	WT_BFA			0,00E+00
	WT_ABA	WT_ABABFA			9,84E-01
	WT_ABA	*big3*_unt			0,00E+00
	WT_ABA	*big3*_ABA			1,00E+00
	WT_ABA	*big3*_BFA			0,00E+00
	WT_ABA	*big3*_ABABFA			0,00E+00
	WT_BFA	WT_ABABFA			0,00E+00
	WT_BFA	*big3*_unt			1,00E+00
	WT_BFA	*big3*_ABA			0,00E+00
	WT_BFA	*big3*_BFA			9,28E-01
	WT_BFA	*big3*_ABABFA			9,99E-01
	WT_ABABFA	*big3*_unt			0,00E+00
	WT_ABABFA	*big3*_ABA			8,60E-01
	WT_ABABFA	*big3*_BFA			0,00E+00
	WT_ABABFA	*big3*_ABABFA			0,00E+00
	*big3*_unt	*big3*_ABA			0,00E+00
	*big3*_unt	*big3*_BFA			7,38E-01
	*big3*_unt	*big3*_ABABFA			9,65E-01
	*big3*_ABA	*big3*_BFA			0,00E+00
	*big3*_ABA	*big3*_ABABFA			0,00E+00
	*big3*_BFA	*big3*_ABABFA			1,00E+00
Figure 4i	WT_unt	WT_ABA	Non parametric Tukey	15	1,24E-08
	WT_unt	WT_BFA			9,68E-01
	WT_unt	WT_ABABFA			1,43E-03
	WT_unt	*big3*_unt			1,00E+00
	WT_unt	*big3*_ABA			5,50E-05
	WT_unt	*big3*_BFA			2,23E-01
	WT_unt	*big3*_ABABFA			9,69E-01
	WT_ABA	WT_BFA			6,93E-07
	WT_ABA	WT_ABABFA			1,75E-02
	WT_ABA	*big3*_unt			1,38E-08

	WT_ABA	*big3*_ABA			1,00E+00
	WT_ABA	*big3*_BFA			6,19E-11
	WT_ABA	*big3*_ABABFA			1,64E-10
	WT_BFA	WT_ABABFA			5,45E-02
	WT_BFA	*big3*_unt			9,54E-01
	WT_BFA	*big3*_ABA			1,93E-03
	WT_BFA	*big3*_BFA			5,22E-02
	WT_BFA	*big3*_ABABFA			5,55E-01
	WT_ABABFA	*big3*_unt			3,03E-03
	WT_ABABFA	*big3*_ABA			4,97E-01
	WT_ABABFA	*big3*_BFA			3,10E-05
	WT_ABABFA	*big3*_ABABFA			6,26E-04
	*big3*_unt	*big3*_ABA			8,88E-05
	*big3*_unt	*big3*_BFA			3,71E-01
	*big3*_unt	*big3*_ABABFA			9,93E-01
	*big3*_ABA	*big3*_BFA			6,55E-08
	*big3*_ABA	*big3*_ABABFA			4,50E-05
	*big3*_BFA	*big3*_ABABFA			8,20E-01
Suppl Fig. 3b	WT	*esb1*	Non parametric Tukey	44	0,00E+00
	WT	*casp1casp3*			0,00E+00
	WT	*sgn3*			1,23E-01
	WT	*esb1sgn3*			8,45E-02
	WT	*casp1casp3sgn3*			1,00E+00
	esb1	*casp1casp3*			3,45E-03
	esb1	*sgn3*			0,00E+00
	esb1	*esb1sgn3*			6,66E-16
	esb1	*casp1casp3sgn3*			0,00E+00
	casp1casp3	*sgn3*			8,74E-12
	casp1casp3	*esb1sgn3*			1,22E-08
	casp1casp3	*casp1casp3sgn3*			8,55E-15
	sgn3	*esb1sgn3*			9,99E-01
	sgn3	*casp1casp3sgn3*			3,18E-01
	esb1sgn3	*casp1casp3sgn3*			2,09E-01
	WT	*esb1*	Non parametric Tukey	4	0,00E+00
	WT	*casp1casp3*			0,00E+00
	WT	*sgn3*			1,00E+00
	WT	*esb1sgn3*			9,67E-01
	WT	*casp1casp3sgn3*			1,00E+00
	esb1	*casp1casp3*			8,28E-01
	esb1	*sgn3*			0,00E+00
	esb1	*esb1sgn3*			0,00E+00

esb1	*casp1casp3sgn3*	0,00E+00
casp1casp3	*sgn3*	0,00E+00
casp1casp3	*esb1sgn3*	0,00E+00
casp1casp3	*casp1casp3sgn3*	0,00E+00
sgn3	*esb1sgn3*	9,67E-01
sgn3	*casp1casp3sgn3*	1,00E+00
esb1sgn3	*casp1casp3sgn3*	9,67E-01

*Data from Figure 3f were statistically analyzed together with data from Figure 3c
