# Supplementary material for: Extracellular vesiculo-tubular structures associated with suberin deposition in plant cell walls
Source: Nat Commun. 2022 Mar 18;13:1489. doi: 10.1038/s41467-022-29110-0 (PMC8933581; doi:10.1038/s41467-022-29110-0)
Supplement: Supplementary file 2 — Description of Additional Supplementary Files [file 41467_2022_29110_MOESM2_ESM.pdf]

Description of Additional Supplementary Files

File name: Supplementary Movie 1

Description: **3D reconstruction of the PM and its EVBs in a chemically-fixed *lotr2/exo70a1* mutant.** Travelling in a series of 40 nm FIB-SEM slices through a Z volume of 10 µm showing the high number of extracellular vesicular-tubules containing bodies (EVBs) in an endodermal suberizing cell of a *lotr2/exo70a1* mutant at 2 mm from the root tip. The 3D model in yellow highlight the PM and its EVBs. The overview picture is shown in the Fig. 1D, some screen shots in Supplementary Fig. 1B and orthogonal views in Supplementary Fig. 1C.

File name: Supplementary Movie 2

Description: **3D reconstruction of one EVB in a chemically-fixed *lotr2/exo70a1* mutant.** Travelling in a series of 0.77 nm optical tomography slices through a Z volume of 191 nm showing the high number of interconnected vesicular-tubules inside one EVB in an endodermal suberizing cell of a *lotr2/exo70a1* mutant at 2 mm from the root tip. The 3D model in yellow highlight one EVB. A single optical tomography slice and an overview picture of the model is shown in the Fig.2A.

File name: Supplementary Movie 3

Description: **3D reconstruction of one EVB in a chemically-fixed WT.** Travelling in a series of 0.38 nm optical tomography slices through a Z volume of 162 nm showing one EVB in an endodermal suberizing cell from a WT root in the suberizing zone. Serie of three optical sections is shown in Fig.2B.

File name: Supplementary Movie 4

Description: **3D reconstruction of one EVB in a chemically-fixed WT.** Travelling in a series of 0.38 nm optical tomography slices through a Z volume of 148 nm showing one EVB and the growing suberin lamellae in an endodermal suberizing cell from a WT root in the suberizing zone. Serie of three optical sections is shown in Fig.2C.

File name: Supplementary Movie 5

Description: **Tomogram of one EVB in a cryofixed *lotr2/exo70a1* mutant.** Tilt series (-60.2°, +54.8°) through a Z volume of 174 nm in an endodermal suberizing cell of a *lotr2/exo70a1* mutant at 2 mm from the root tip.

File name: Supplementary Movie 6

Description: **3D reconstruction of one EVB in a cryofixed *lotr2/exo70a1* mutant.** Travelling in a series of 0.38 nm optical tomography slices through a Z volume of 174 nm showing one EVB in an endodermal suberizing cell of a *lotr2/exo70a1* mutant at 2 mm from the root tip. Serie of three optical sections and the model is show in Fig.2D.

File name: Supplementary Movie 7

Description: **3D reconstruction of one EVB in a cryofixed *lotr2/exo70a1* mutant.** Travelling in a series of 0.38 nm optical tomography slices through a Z volume of 174 nm showing one EVB in an endodermal

suberizing cell of a *lotr2/exo70a1* mutant at 2 mm from the root tip. The 3D model in yellow highlight one EVB and its ginger root like structure. Serie of three optical sections and the model is show in Fig.2D.

File name: Supplementary Movie 8

Description: **Tomogram of one EVB in a cryofixed *esb1* mutant.** Tilt series (-60.2°, +59.8°) through a Z volume of 140 nm showing one EVB in an endodermal suberizing cell of an esb1 mutant at 2 mm from the root tip.

File name: Supplementary Movie 9

Description: **3D reconstruction of one EVB in a cryofixed *esb1* mutant.** Travelling in a series of 0.576 nm optical tomography slices through a Z volume of 140 nm showing one EVB in an endodermal suberizing cell of an esb1 mutant at 2 mm from the root tip. Serie of three optical sections is show in Fig.2E.
